# Bumped pomalidomide-based PROTACs

**DOI:** 10.1038/s42004-024-01125-2

**Published:** 2024-02-26

**Authors:** Huijuan Guo

**Affiliations:** Communications Chemistry, https://www.nature.com/commschem

## Abstract

Pomalidomide is an E3 ligase recruiter exploited by PROTACs to degrade target proteins, but its application is hampered by the off-target degradation of other vital endogenous zinc finger (ZF) proteins. Now, the off-target ZF binding of pomalidomide-based PROTACs is evaluated by a high-throughput imaging screening platform, and minimization of off-target degradation as well as enhanced potency are achieved through selective functionalization at the C5 position of the phthalimide ring.

Pomalidomide is an FDA-approved drug for the treatment of multiple myeloma based on its binding to an E3 ligase component cereblon (CRBN) *via* its glurarimide ring. It also works as a molecular glue that can simultaneously bind to the CRBN *via* its glurarimide ring and human Cys2-His2 (C2H2) zinc finger (ZF) domain *via* its phthalimide ring. In addition, pomalidomide can be developed as a hetero-bifunctional proteolysis-targeting chimera (PROTAC), by appending to the target protein binder through the C4/C5 position of the phthalimide ring to generate a molecular chimera that induces proximity between the CRBN ligase complex and target proteins. This proximity induction between CRBN and ZF proteins or target proteins triggers the latter’s ubiquitination and degradation by proteasome. However, pomalidomide can degrade a wide range of human C2H2 ZF proteins^1^, which causes the off-target degradation of ZF proteins that have vital roles in health and disease.

Now, a team led by Amit Choudhary at Broad Institute of Harvard and MIT in the USA has developed a robust high-throughput imaging platform that profiles the off-target degradation propensities of pomalidomide-based PROTACs towards ZF domains. They study a library of pomalidomide analogues with varying functional groups, and rationally modify the pomalidomide to reduce off-target effects and enhance the potency of an anaplastic lymphoma kinase (ALK) oncoprotein-targeting PROTAC (Fig. 1) (10.1038/s41557-023-01379-8)^2^.

**Fig. 1:**
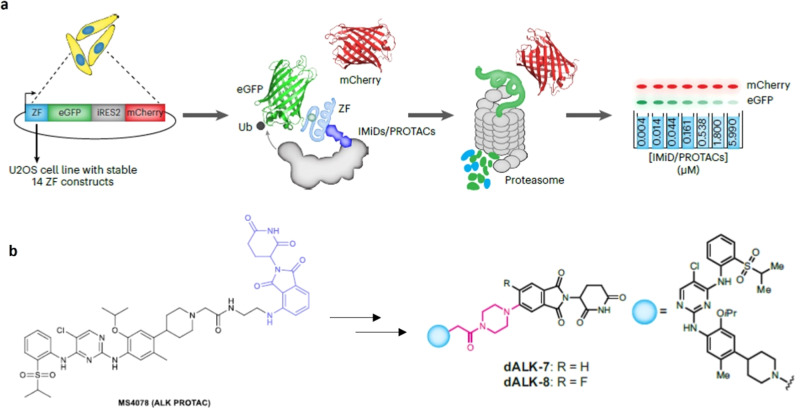
Bumped pomalidomide-based PROTACs. **a** Design principle of a high-throughput imaging-based assay for evaluating the off-target zinc finger degradation of pomalidomide-based PROTACs; **b** original structure of ALK PROTAC (MS4078) and bumped ALK PROTAC (dALK-7 and -8). Adapted from *Nature Chemistry* (2023) 10.1038/s41557-023-01379-8.

Currently, mass spectrometry-based proteomics is the most prevalent technique used to assess off-target degradation, but it usually runs at a high cost, is challenging to implement, has limited high-throughput capabilities, and lacks sensitivity to low-abundance proteins. The team previously developed an automatic imaging-based high-throughput assay to screen the pomalidomide-induced human C2H2 zinc finger degrome^1^, where ZF degrons were genetically inserted into a degradation reporter system to monitor the decrease in fluorescence of a panel of various GFP-tagged ZF domains, comparing with untagged mCherry upon compound treatment. This reporter-based method is not limited by cell-type-specific expression levels of analyte proteins, nor by the accessibility to ZFs in protein complexes, and the team posited that it may display enhanced sensitivity over MS-based methods for the detection of pomalidomide-sensitive ZF protein degradation.

Using this imaging-based platform, the researchers found that nearly all of the profiled pomalidomide-based PROTACs exhibited off-target degradation of various ZF proteins. Based on the co-crystal structure of the DDB1–CRBN–pomalidomide complex bound to transcription factor IKZF1^1^, they surmised that increasing the size of substituents at the C4 and /or C5 position of the phthalimide ring, and masking the hydrogen bond donors, could potentially ‘bump off’ and disrupt the ternary complex of the ZF domains with CRBN, while maintaining pomalidomide’s interaction with CRBN through the glutarimide ring. The team therefore constructed a library of rationally designed pomalidomide analogues, synthesized them in pairs at the C4 and C5 positions, with fluoro substitution at the C6 position, and tested the library to derive structure–activity relationships using the developed off-target profiling platform. Based on their findings, they generated PROTACs with piperazine and 2,6-diazaspiro[3.3]heptane substitution at the C5 position and found that these target ALK with reduced off-target ZF protein degradation and enhanced on-target potencies.

“The direct application of this platform is profiling the off-target effects of PROTACs. Beyond that, we describe approaches to reduce off-targets of PROTACs and provide a rich dataset that can assist with rational design of molecular glues and PROTACs,” comments Choudhary.
